# Patients with non-Sjögren’s sicca report poorer general and oral health-related quality of life than patients with Sjögren’s syndrome: a cross-sectional study

**DOI:** 10.1038/s41598-020-59078-0

**Published:** 2020-02-07

**Authors:** B. Tashbayev, T. Garen, Ø. Palm, X. Chen, B. B. Herlofson, A. Young, L. H. Hove, M. Rykke, P. B. Singh, L. A. Aqrawi, Ø. A. Utheim, T. P. Utheim, J. L. Jensen

**Affiliations:** 1 Department of Oral Surgery and Oral Medicine, Faculty of Dentistry, University of Oslo, Oslo, Norway; 2 Department of Rheumatology, Oslo University Hospital, Oslo, Norway; 3 Department of Cariology and Gerodontology, Faculty of Dentistry, University of Oslo, Oslo, Norway; 4 Department of Ophthalmology, Oslo University Hospital, Oslo, Norway; 5 Department of Oral Biology, Faculty of Dentistry, University of Oslo, Oslo, Norway; 6 Department of Medical Biochemistry, Oslo University Hospital, Oslo, Norway

**Keywords:** Salivary gland diseases, Health care, Quality of life

## Abstract

Understanding the impact of the disease on quality of life is crucial in patient management. In this cross-sectional study, general and oral health-related quality of life questionnaires, and thorough examinations of oral and ocular dryness were performed in age- and sex-matched patients with primary Sjögren’s syndrome (pSS group), non-Sjögren’s syndrome sicca (non-SS group) and healthy controls. General and oral health-related quality of life were investigated with the 36-Item Short Form Health Survey and the 14-Item Oral Health Impact Profile questionnaires, respectively. Subjective symptoms of xerostomia and ocular dryness were recorded using the Summated Xerostomia Inventory and Ocular Surface Disease Index, respectively. Clinical examinations included evaluation of clinical oral dryness scores, candida counts, unstimulated and stimulated saliva secretory rates, tear osmolarity, tear film break-up time, Schirmer I test and ocular surface staining. Both patient groups had pronounced signs and symptoms of xerostomia and ocular dryness. Even though the non-SS patients had less severe clinical signs than the pSS patients, they demonstrated much poorer general and oral health-related quality of life. In conclusion, non-SS patients require more attention in order to improve their quality of life.

## Introduction

Sjögren’s syndrome (SS) is a systemic, autoimmune connective tissue disease presenting a wide range of sicca symptoms, mainly dry mouth and dry eyes that result from permanently impaired salivary and lacrimal gland function^1^. Sjögren’s syndrome is also associated with extraglandular renal, pulmonary, and neurological manifestations in about 30% of patients^2^. Around 5% of patients may develop lymphoma which is the most severe complication^2,3^. Sjögren’s syndrome is defined as primary SS (pSS) when occurring in the absence of an underlying rheumatic disorder, and as secondary SS (sSS) when associated with another connective tissue disorder^4,5^. The large and diverse group of patients that have sicca symptoms consists of patients with various disorders who receive medical treatments that cause hypofunction of salivary and/or lacrimal glands. However, some patients have sicca symptoms in the absence of known diseases or medication, but without the distinctive features of pSS, namely Ro/SSA and/or La/SSB autoantibodies and lymphocyte infiltration in their minor salivary glands^6^. In the present paper, this diverse group of patients was named the non-SS group, in order to highlight the fact that although they present with SS-like symptomatology they do not fulfil the diagnostic criteria for SS^7–20^.

The oral manifestations of SS can include subjective symptoms of dry mouth (xerostomia), burning sensation on the tongue, reduced sense of taste, and difficulties in swallowing food^21^. Clinical findings of dry mouth include dry and fissured tongue, lack of saliva pool and bubbly saliva, tooth decay, and candidiasis^22^. The subjective symptoms of dry eyes in SS can manifest as reduced vision, ocular discomfort, scratchiness, and pain in the eyes. Clinical findings of dry eyes may involve damage to the ocular surface - the cornea, conjunctiva, and eyelids^23,24^. The prevalence of pSS shows a strong female propensity, and depending on the criteria used for classification, is estimated to range between 0.05% and 2.7%^25^. The complexity, symptoms and complications of pSS are shown to have a clear negative effect on the quality of life (QoL) of these patients^26–30^. However, the QoL in patients with the non-SS sicca complex has not been as extensively studied. We have previously shown a comparatively high symptom burden in non-SS as compared to pSS patients, despite more pronounced clinical findings in the pSS patients^20^. Therefore, studies on both general health-related QoL (GHRQoL) and oral health-related QoL (OHRQoL), comparing patients with pSS and non-SS, are of paramount importance for achieving overall understanding of the disease burden of patients with non-SS.

In this study, our objective was to investigate the GHRQoL and OHRQoL in age- and sex-matched groups of patients with pSS, the non-SS group, and healthy controls. We also explored correlations between QoL and subjective and clinical findings of oral and ocular dryness.

## Methods

### Participants

This cross-sectional study was carried out in collaboration with the Department of Rheumatology, Oslo University Hospital (OUH); the Dry Mouth Clinic, Faculty of Dentistry, University of Oslo; and the Norwegian Dry Eye Clinic. The Norwegian Regional Committee for Medical and Health Research Ethics approved the study protocol (REK 2015/363), and the study was performed in compliance with the tenets of the Declaration of Helsinki. Written informed consent was obtained from all subjects prior to participation in the study that was performed between August 2015 and September 2018.

All participants were females aged 30–80 years, had no other diseases that could explain sicca symptomatology and did not use multiple medications influencing saliva and tear production. Sixty patients with pSS (pSS group), 22 patients with non-SS sicca (non-SS group), and 43 healthy subjects (control group) were recruited for the study. All patients with pSS fulfilled the classification criteria established by the American-European Consensus Group (AECG)^8^ and had serum-positive anti-SSA antibodies. The non-SS group included age- and sex-matched patients with symptoms of ocular or oral dryness and impaired salivary and/or lacrimal gland function (sicca complex). The non-SS patients had neither positive anti-SSA/SSB serum antibodies nor positive salivary gland biopsies, and thus did not fulfill the AECG criteria. Importantly, all non-SS patients were recruited based on the absence of other diseases or use of multiple medications influencing saliva and tear production. Patients using multiple medications and having a systemic disease that could involve hypofunction of salivary and lacrimal glands were not included. The non-SS group is a distinctive group of patients who are referred to rheumatologists for SS workup but in the end lack enough evidence to classify them as patients with SS. The control group comprised 43 age- and sex-matched healthy female participants. The inclusion criteria for the healthy controls were absence of symptoms of dryness of the mouth or eyes, and absence of any disorders with oral or ocular involvement. Potential participants with a history of surgical procedures that might affect secretion from the salivary or lacrimal glands were excluded. Table 1 lists the characteristics of the participants.

**Table 1:** Description of the study population.

Characteristic	pSS group (n = 60)	Non-SS group (n = 22)	Control group (n = 43)	p-value (Intergroup comparison)
Age (y)	53.6 ± 13.2	52.0 ± 10.4	49.2 ± 13.8	0.244
Range	26–75	34–76	20–79	
Height (cm)	169.0 ± 6.5	166.7 ± 5.3	167.8 ± 4.9	0.246
Range	153–182	158–178	157–185	
Weight (kg)	71.4 ± 13.4	72.3 ± 16.2	65.2 ± 10.3	0.035
Range	49–120	51–120	50–90	
Ethnicity				0.079
*Scandinavian*	57	20	34	
*West European*	1	0	1	
*East European*	1	0	4	
*African*	1	0	0	
*South American*	0	0	1	
*Asian*	0	2	1	
*Oceanian*	0	0	2	
Education				0.021
*Elementary*	6 (10%)	1 (5%)	1 (2%)	
*Secondary*	19 (32%)	10 (45%)	7 (17%)	
*Higher*	35 (58%)	11 (50%)	35 (81%)	
SXI-D	11.8 ± 2.5^a,e^	12.3 ± 1.9^b^	6.2 ± 0.4	<0.001
*Candida* score	1.4 ± 1.1^a,e^	0.8 ± 0.9^f^	0.5 ± 0.8	<0.001
CODS	5.0 ± 1.9 ^a,e^	4.2 ± 2.1^b^	0.6 ± 0.9	<0.001
UWS (ml/min)	1.3 ± 1.2 ^a,e^	1.7 ± 1.2 ^b^	4.5 ± 2.6	<0.001
SWS (ml/min)	3.5 ± 2.8^a,e^	4.8 ± 1.7^b^	7.7 ± 3.5	<0.001
OSDI	31.7 ± 18.9^a,c^	51.7 ± 24.0^b^	4.1 ± 6.5	<0.001
Tear osmolarity (mOsm/L)	328.2 ± 23.2^d,e^	326.7 ± 22.3^f^	314.1 ± 22.7	0.04
TFBUT (seconds)	2.3 ± 2.1^a,e^	3.1 ± 2.1^b^	5.9 ± 4.0	<0.001
Schirmer test (mm/5 min)	5.7 ± 5.6^a,c^	12.9 ± 9.6^f^	17.0 ± 11.1	<0.001
Ocular surface staining	3.2 ± 2.6^a,c^	1.3 ± 1.2^f^	0.8 ± 1.2	<0.001

^a^Significant difference between pSS and controls, p < 0.01.

^b^Significant difference between non-SS and controls, p < 0.01.

^c^Significant difference between pSS and non-SS, p < 0.01.

^d^Significant difference between pSS and non-SS, p < 0.05.

^e^Difference between pSS and non-SS not significant at p < 0.05.

^f^Difference between non-SS and controls not significant at p < 0.05.

### Evaluation of general health related quality of life

The GHRQoL in all groups was evaluated using the 36-Item Short Form Health Survey (SF-36) (Norwegian version; Quality Metric Inc., Lincoln, RI). The SF-36 is a multipurpose, generic health survey instrument developed by the Medical Outcomes Study^31^. The questionnaire consists of 36 items measuring eight dimensions of QoL (physical functioning, role physical, bodily pain, general health, vitality, social functioning, role emotional, mental health). The response from each dimension is converted to a composite score of 0–100, where 0 implies the worst possible GHRQoL status and 100 the best. In addition to the eight dimensions, two summated scores have been defined: the physical component summary (PCS) and the mental component summary (MCS). The PCS and MCS are aggregated measures of the eight dimensions and are used to evaluate physical and mental health, respectively^32^. The validity and reliability of the SF-36 have been established in several health surveys of different patient populations^33–35^ including patients with pSS^36–38^.

### Evaluation of oral health related quality of life

Oral Health-related Quality of Life is defined as “the absence of negative impacts of oral conditions on social life and a positive sense of dentofacial self-confidence” and has been measured with various instruments^39^. One of the most frequently used questionnaires is the Oral Health Impact Profile-14 (OHIP-14) questionnaire, a short form of the original OHIP-49. It was developed by Slade *et al*.^40^ with the aim of providing a comprehensive measure of self-reported dysfunction, discomfort, and disability attributed to the oral condition, and the questionnaire has acceptable validity and reliability^40,41^. The questionnaire also aims to retrieve information on impacts related to oral conditions in general, rather than impacts that may be attributed to specific oral disorders or syndromes. The OHIP-14 consists of 14 statements rephrased as questions and organized into seven dimensions [functional limitation (Q1 + Q2), physical pain (Q3 + Q4), psychological discomfort (Q5 + Q6), physical disability (Q7 + Q8), psychological disability (Q9 + Q10), social disability (Q11 + Q12), handicap (Q13 + Q14)], and addresses various aspects of oral health^42^. Respondents were instructed to indicate their experience with a particular problem on a five-point Likert scale and are given the following response categories: “Never”, “Hardly ever”, “Occasionally”, “Fairly often”, and “Very often”. The responses are scored using a scale of 0–4 (0 = never; 1 = hardly ever; 2 = occasionally; 3 = fairly often; 4 = very often). The sum of the scores ranges 0–56, where a low score indicates high OHRQoL. In this study we used the Norwegian version of the OHIP-14^43^.

### Oral examination

The examination protocol was published earlier, utilizing a smaller dataset^20^. Briefly, all participants were instructed to refrain from eating, drinking, and smoking 1 hour prior to their appointment at the Dry Mouth Clinic. Participants were asked to respond to the five statements that form the Summated Xerostomia Inventory-Dutch version (SXI-D)^44^. The SXI-D is a shortened version of the Xerostomia Inventory (XI) questionnaire used to determine the severity of xerostomia^45^. The SXI-D sum score can range from 5 to 15, where the maximum sum score is indicative of (extremely) severe problems related to dry mouth.

The participants underwent a thorough oral clinical examination, including collection of unstimulated (UWS) and chewing-stimulated whole saliva (SWS). An objective score for oral dryness was obtained using the Clinical Oral Dryness Score (CODS)^46^. The CODS is determined from 10 different features of oral dryness, and each positive feature scores 1 point for a total of 0 to 10, with higher scores indicating more severe oral dryness. The presence of oral *Candida* was tested by rubbing a sterile cotton swab over two oral mucosal sites: the left cheek and the anterior part of the tongue. Samples were inoculated on Sabouraud dextrose agar plates, incubated for 4 days at 37 °C, and growth was scored semi-quantitatively: 0 no growth; 1 = 1–9 colonies (minimal growth); 2 = 10–29 colonies (moderate growth); 3 > 30 colonies (severe growth)^47^.

### Eye examination

All participants underwent subjective and objective dry eye examinations at the Norwegian Dry Eye Clinic. The subjective evaluation was performed using the Ocular Surface Disease Index (OSDI)^48^. The OSDI is a 12-item questionnaire designed to provide a rapid assessment of the symptoms of ocular irritation consistent with dry eye disease (DED) and their impact on vision-related functioning. The OSDI scale ranges 0–100, with higher scores representing greater disability due to eye symptoms. The overall OSDI score defines non-DED (0–12 points), and mild (13–22 points), moderate (23–32 points), and severe DED (33–100 points)^49^.

After subjective evaluation of DED with the questionnaires, the participants underwent tear osmolarity measurement using the TearLab Osmolarity System (TearLab, San Diego, CA)^50^, tear film break-up time (TFBUT) measurement^51,52^, assessment of corneal sensitivity^53^, ocular surface staining (OSS)^54^, and tear production rates with the Schirmer test^51^.

### Statistical analyses

The statistical analyses were performed with the commercial software SPSS for Windows, version 22 (IBM, Chicago, IL). The normality of variables was verified by the Shapiro–Wilk tests. The means of all data for the QoL, and oral and ocular measurements of the three groups were compared. One-way analysis of variance (ANOVA) was used in the intergroup comparison of parameters with normal distribution, while the Kruskal-Wallis H test was used for parameters with non-normal distribution. Correlation between variables was checked using the Pearson correlation and Spearman rank analyses. Regression analyses with adjustment for potential confounders were performed to test for possible relationships between clinical and QoL parameters. For intergroup comparison, p < 0.017 (due to post hoc analysis) was considered statistically significant; p < 0.05 was used for other analyses. The results are presented as the mean ± standard deviation (SD).

## Results

### Participant characteristics, oral and ocular dryness examination

Assessment of the GHRQoL and OHRQoL, and comprehensive evaluation of oral dryness and DED were carried out in the three groups; pSS, non-SS, and control.

There was no missing data. The characteristics of the three study groups are shown in Table  1 (Appendix 1). Similar to our previous publication that involved fewer participants^20^, the non-SS group reported higher scores in response to the SXI-D questionnaire than the pSS group, indicating a more pronounced subjective feeling of oral dryness. In the objective examinations, the non-SS group appeared to have better oral health, with lower scores for *Candida* count and CODS, and higher saliva secretory rates. The non-SS participants had a higher mean OSDI score than the pSS participants, indicating more severe dry eye symptoms. In contrast, the non-SS group had less severe ocular objective clinical findings than the pSS group, with longer TFBUT, higher Schirmer test value, and less OSS. Notably, the Schirmer test results of the non-SS participants exceeded the normal threshold value of 10 mm/5 min. An overview of medication use in the three groups is given in Appendix 1 and shows that the only statistically significant difference between the patient groups was that a greater percentage of the non-SS patients used hypnotics and sedatives; 27% of the non-SS patients vs 5% of pSS patients (p = 0.04).

### Evaluation of general health-related quality of life

SF-36 evaluation of GHRQoL revealed overall that the pSS and non-SS groups had significantly reduced QoL compared to the control group (Table 2). The differences between the groups for all eight dimensions and for the two component scores, PCS and MCS, were statistically significant. The non-SS group had significantly lower mean scores than the pSS group for the dimensions of *role physical* (13.6 ± 27.5), *vitality* (21.1 ± 18.3), and *general health* (31.5 ± 18.8). The pSS and non-SS groups had similar mental health dimension scores.

**Table 2:** Comparison of GHRQoL results measured with the SF-36.

	pSS group (n = 60)	Non-SS group (n = 22)	Control group (n = 43)	p-value (intergroup comparison)
	Mean ± SD	Mean ± SD	Mean ± SD	
Physical functioning	74.7 ± 21.5^a,d^	63.4 ± 23.3^b^	96.5 ± 6.9	<0.001
Role physical	51.3 ± 38.7^a,c^	13.6 ± 27.5^b^	90.4 ± 24.1	<0.001
Bodily pain	60.6 ± 22.3^a,c^	40.9 ± 30.1^b^	89.1 ± 16.3	<0.001
General health	45.2 ± 22.0^a,d^	31.5 ± 18.8^b^	86.1 ± 14.0	<0.001
Vitality	37.9 ± 24.0^a,c^	21.1 ± 18.3^b^	70.4 ± 17.8	<0.001
Social functioning	64.0 ± 25.6^a,e^	52.8 ± 24.4^b^	95.5 ± 9.3	<0.001
Role emotional	69.3.3 ± 38.1^a,e^	56.1 ± 45.3^b^	96.6 ± 10.2	<0.001
Mental health	76.1 ± 16.9^a,e^	74.2 ± 18.3^b^	86.2 ± 12.6	0.004
PCS	41.4 ± 10.2^a,c^	31.5 ± 10.2^b^	56.8 ± 5.0	<0.001
MCS	46.2 ± 10.3^a,e^	44.1 ± 11.6^b^	55.1 ± 6.2	<0.001

^a^Significant difference between pSS and controls, p < 0.01.

^b^Significant difference between non-SS and controls, p < 0.01.

^c^Significant differen^c^e between pSS and non-SS, p < 0.01.

^d^Significant difference between pSS and non-SS, p < 0.05.

^e^Difference between pSS and non-SS not significant at p < 0.05.

### Evaluation of oral health-related quality of life

Comparison of the summated mean scores of the OHRQoL showed that the non-SS group had the highest total score (18.6 ± 13.9), followed by the pSS group (13.5 ± 10.5, p = 0.035) and the controls (2.2 ± 2.9, p < 0.001). Significant differences were observed for all seven dimensions between all groups. Table 3 summarizes these findings.

**Table 3:** Comparison of the OHRQoL results measured with the OHIP-14.

	pSS group (n = 60)	Non-SS group (n = 22)	Control group (n = 43)	p-value (intergroup comparison)
	Mean ± SD	Mean ± SD	Mean ± SD	
Functional limitations (Q1 + Q2)	1.9 ± 1.9^a,c^	3.9 ± 2.1^b^	0.2 ± 0.5	<0.001
Physical pain (Q3 + Q4)	3.2 ± 2.1^a,e^	3.5 ± 2.2^b^	0.8 ± 1.1	<0.001
Psychological discomfort (Q5 + Q6)	2.8 ± 2.7^a,e^	3.4 ± 2.8^b^	0.4 ± 0.8	<0.001
Physical disability (Q7 + Q8)	1.1 ± 1.5^a,e^	1.6 ± 2.6^b^	0.1 ± 0.4	<0.001
Psychological disability (Q9 + Q10)	1.9 ± 1.9^a,d^	3.0 ± 2.6^b^	0.4 ± 0.8	<0.001
Social disability (Q11 + Q12)	0.9 ± 1.3^a,e^	1.4 ± 2.6^b^	0.09 ± 0.4	=0.001
Handicap (Q13 + Q14)	1.7 ± 1.8^a,e^	1.9 ± 2.6^b^	0.09 ± 0.4	<0.001
Total score (OHIP-14)	13.5 ± 10.5^a^	18.6 ± 13.9^b^	2.2 ± 2.9	<0.001

^a^Significant difference between pSS and controls, p < 0.01.

^b^Significant difference between non-SS and controls, p < 0.01.

^c^Significant differen^c^e between pSS and non-SS, p < 0.01.

^d^Significant difference between pSS and non-SS, p < 0.05.

^e^Difference between pSS and non-SS not significant at p < 0.05.

### Correlation analyses: Quality of life, oral and ocular dryness

We found several significant correlations between the subjective (SF-36) and objective parameters for oral and ocular dryness in all groups (Appendix 2 and 3). In the pSS group, there were low to moderate negative correlations between the dimensions and component scores of the SF-36 and the subjective feeling of oral and ocular dryness as measured with the SXI-D and OSDI. We did not find any significant correlations between the SF-36 data and the objective findings of oral and ocular dryness. In the non-SS group, one SF-36 dimension, i.e., *role physical*, correlated moderately with ocular surface staining. In the control group, *physical functioning* correlated negatively with OSDI and tear osmolarity, while *social functioning* correlated with OSDI and *Candida* score. Interestingly, *vitality* correlated strongly with the SXI-D.

In the pSS group, we found several significant correlations between the OHIP-14 summary score and oral dryness. The *Candida* score correlated most strongly with the OHIP-14 sum score (r = 0.79, p < 0.05), followed by the SXI-D (r = 0.549, p < 0.001). Furthermore, the subjective feeling of oral dryness reported with the SXI-D correlated moderately with PCS (r = −0.406, p < 0.001) and weakly with MCS (r = 0.325, p < 0.001). Similarly, the subjective feeling of ocular dryness measured with the OSDI correlated moderately with PCS (r = −0.45, p < 0.001) and weakly with MCS (r = −0.287, p < 0.001).

The non-SS group did not demonstrate significant correlations between the SF-36 results and the other clinical data obtained. Interestingly, also the OHIP-14 results did not show any correlation with the results for oral dryness in this group. However, there were moderate negative correlations between the OHIP-14 summary score and the OSDI (r = −0.406), TFBUT (r = −0.450), ocular staining (r = −0.440), and a weak correlation with tear osmolarity (r = −0.325), all at p < 0.001.

## Discussion

The present study demonstrates that the patients with pSS had significantly reduced QoL, and that patients with non-SS sicca had even more reduced QoL than patients with pSS. The patients with non-SS sicca had considerably lower scores in all dimensions of the SF-36. Evaluation of the OHRQoL revealed a similar pattern; pSS patients had significantly lower scores than controls and the scores of the non-SS patients were significantly more decreased in all dimensions of the questionnaire. Patients with non-SS sicca, who would appear to have similar symptoms to patients with pSS, traditionally receive very little attention, as they do not fulfil the classification criteria for SS and have less profound clinical findings. Our results underline the necessity of providing optimal care for both patients with pSS and patients with non-SS sicca.

Interestingly, despite having significantly less pronounced clinical characteristics of dry mouth and slightly better saliva production rates, the non-SS sicca group had more subjective symptoms of oral dryness. The non-SS sicca group also had more subjective symptoms of ocular dryness, despite less pronounced clinical characteristics of ocular dryness (higher tear production levels, more stable tear film, less damaged ocular surfaces and lower tear osmolarity levels) compared to patients with pSS. In other words, although the pSS patients had more prominent clinical features of oral and ocular dryness, reduced saliva secretion, and reduced tear production, they reported having a better quality of life than the non-SS sicca group. However, while no special correlations were observed between the various parameters and the SF-36 scores in the non-SS sicca group, there were a few significant correlations between the OHIP-14 sum scores and subjective as well as clinical dry eye findings. In the pSS group, significant correlations were demonstrated between the two composite components of SF-36 and oral and ocular dryness. Significant correlations were also demonstrated between the OHIP-14 sum score and dry eye findings in the pSS group.

### General health-related quality of life

Patients with medical conditions may have a compromised general quality of life as a result. In our study, the pSS and non-SS groups had significantly reduced GHRQoL compared to the control group. The non-SS group had the lowest scores in all eight dimensions of the SF-36. Several earlier studies have previously demonstrated that patients with pSS have lower GHRQoL than healthy controls^55–58^. There are studies that have investigated QoL in patients with other systemic disorders such as rheumatoid arthritis (RA)^36^ and systemic lupus erythematosus^59,60^. Strömbeck *et al*. reported that patients with RA score lower in the dimensions *physical function* and *role physical* compared to patients with pSS. However, few studies have focused on the general QoL in patients with non-SS sicca. Cho *et al*. reported that patients with non-SS sicca had SF-36 scores similar to that of patients with pSS^26^. Champey and co-workers compared the QoL in pSS and non-SS sicca patients^29^. They concluded that despite their findings indicating a lower QoL in both patient groups as compared to healthy controls, the two patient groups were not different^29^. Milin *et al*. reported that patients with non-SS sicca had similarly low GHRQoL as patients with pSS^61^. A large cross-sectional study that assessed 2401 patients with sicca symptoms (44% with SS and 56% without SS), reported that the non-SS patients had significantly lower PCS and MCS components of SF-36^62^. In that study, the authors only carried out a survey without clinical examinations. In the present study, the non-SS sicca group had significantly lower scores in all eight dimensions as well as in the two composite scores of the SF-36, indicating lower GHRQoL than for the pSS patient and control groups. Scores < 40 on both PCS and MCS are defined as caseness, meaning that the participants have poor QoL requiring treatment or help to improve the QoL^63–65^. Thus, it appears that the pSS group is coping better with the disease in the physical, social, and emotional aspects of daily life than the non-SS group. One reason for this may be that obtaining the specific diagnosis of pSS, even if curable treatment is not available, rather than not to receiving a ‘proper’ diagnosis, as is the case for the non-SS patients, has a positive impact on their life situation. Moreover, the pathogenesis of non-SS is not clear, and we cannot rule out that symptoms except the oral and ocular have impact on the QoL. Regardless, inconsistent results among different studies may be due to differences in disease definition or inclusion criteria.

### Oral health–related quality of life

Oral health is an integral part of general well-being, and activities such as conversing, smiling, and eating are important determinants of daily QoL^66^. Oral health-related quality of life has been studied in patients with pSS^27,67–69^ and other systemic disorders^70–72^. Not surprisingly, using the OHIP-49 questionnaire, Lopez-Jornet *et al*. found that patients with pSS had lower OHRQoL compared to patients with secondary SS (24.15 ± 24.34 vs 10.27 ± 14.65)^27^. Similar findings were reported by McMillan and co-workers^67^. It has been established in several studies that patients with both pSS and sSS suffer from significantly reduced OHRQoL^27,30,67–69,73^. However, research on OHRQoL in patients with non-SS sicca has been very limited. In the present study, the non-SS group had the highest summated OHIP-14 score (18.6 ± 13.9), followed by the pSS group (13.5 ± 10.5) and the controls (2.2 ± 2.9), indicating that the non-SS group had the lowest OHRQoL. The same pattern was observed when the seven dimensions of the OHIP-14 were compared (Table 3). The non-SS group reported lower scores for the dimensions of functional limitations, psychological discomfort, and psychological disability. These findings indicate that poor oral health has a negative psychological impact. This highlights the importance of research in this area.

### Dry mouth and dry eye parameters

Thorough clinical evaluation of dry mouth revealed that the pSS group had more pronounced objective dry mouth findings. Our earlier publication with fewer study participants reported similar results. Interestingly, even though the non-SS group had less pronounced dry mouth signs, patients in the group reported higher scores in response to standard xerostomia questions (SXI-D; non-SS, 12.3 ± 1.9 vs. pSS, 11.8 ± 2.5). A similar pattern was observed in the dry eye examination results. The non-SS group had less prominent dry eye signs and even normal tear production rates, as measured with the Schirmer test, while OSS was slightly higher than that in the controls. However, the subjective feeling of ocular dryness measured with the OSDI was significantly higher in the non-SS group (51.7 ± 24.0) than in the pSS group (31.7 ± 18.9). An OSDI score > 32 indicates severe dry eye problems. It can only be speculated that not having a specific diagnosis may exacerbate the subjective feeling of oral and ocular dryness, as seen in the non-SS subjects.

### Correlation between QoL and clinical findings

Clinicians often have limited tools for studying patients’ QoL in daily practice. Thus, identifying clinical biomarkers that aid understanding of the extent of reduced QoL is of paramount importance in disease management. This might help provide individual tailored care.

In the pSS group, clinical findings and the SF-36 scores did not correlate and similar findings were also reported by Champey *et al*.^29^. However, we found correlations between subjective feeling of oral/ocular dryness and the two components scores of the SF-36. These findings imply that the subjective feeling of oral and ocular dryness can be determinants of reduced GHRQoL.

In the pSS group, OHIP-14 and *Candida score*, SXI correlated strongly. The presence of candidiasis and the subjective feeling of oral dryness can be strong indicators of reduced OHRQoL and may be used by clinicians to obtain better understanding of the disease severity.

The non-SS group did not demonstrate significant correlations between the SF-36 results and the other clinical data obtained. Interestingly, the OHIP-14 results did not show any correlation with the results for oral dryness in this group. The moderate correlation found between objective findings of ocular dryness and OHIP-14 in the non-SS group is hard to explain.

The novelty of the present study is the thorough oral as well as ocular examination combined with GHRQoL, OHRQoL in three different groups, namely pSS, non-SS, and healthy controls. Moreover, strict patient selection and focus on the non-SS group strengthened the study. Furthermore, we report extensive correlation analyses between clinical and QoL data. To our knowledge, no such study has been conducted previously. However, the study has a few limitations. Even though healthy controls should not have subjective feeling of ocular dryness, we found that some of them had dry eye signs as observed during the clinical examination. This can be explained by the high prevalence of asymptomatic DED in the general population. Many patients with sicca symptoms undergo extensive examinations to determine whether they have pSS, including saliva and tear measurements; blood sampling; and evaluations by dentists, physicians, and rheumatologists, but most patients end up without a diagnosis. In theory, the non-SS group can include a variety of diseases presenting with sicca symptomatology. However, in the present study the non-SS group was selected based on very strict inclusion criteria. All patients included in the non-SS group had sicca symptoms and findings for unknown reasons (i.e., not drug or disease related), they were negative for serum autoantibodies, and had a negative biopsy obtained by the same oral and maxillofacial surgeon (JLJ) as the pSS patients. The selection criteria resulted in fewer eligible patients to be included in our study. This fact may make it difficult to generalize the study results for other populations of non-SS patients that are based on a wider definition.

In conclusion, a Norwegian cohort of patients with pSS demonstrated significantly lower GHRQoL and OHRQoL compared to the age- and sex-matched healthy controls, and the patients with non-SS had the poorest GHRQoL and OHRQoL. An important finding of the study is that patients with non-SS may suffer as much, or even more, than patients with a pSS diagnosis. Thus, clinicians should be aware of this fact and provide appropriate care and attention for these patients, with frequent follow-ups and examinations in spite of them not fulfilling the AECG classification criteria.

## Supplementary information

Supplementary material.
