# Supplementary material for: Patients with non-Sjögren’s sicca report poorer general and oral health-related quality of life than patients with Sjögren’s syndrome: a cross-sectional study
Source: Sci Rep. 2020 Feb 7;10:2063. doi: 10.1038/s41598-020-59078-0 (PMC7005680; doi:10.1038/s41598-020-59078-0)
Supplement: Supplementary file 1 — Supplementary material. [file 41598_2020_59078_MOESM1_ESM.docx]

**Patients with non-Sjögren’s sicca report poorer general and oral health-related quality of life than patients with Sjögren’s syndrome: a cross-sectional study**

Tashbayev B^1^, Garen T^2^, Palm Ø^2^, Chen X^1^, Herlofson BB^1^, Young A^3^, Hove LH^3^, Rykke M^3^, Singh PB^1^, Aqrawi LA^1^, Utheim ØA^4^, Utheim TP ^5,6^, Jensen JL^1^

1. Department of Oral Surgery and Oral Medicine, Faculty of Dentistry, University of Oslo, Oslo, Norway.
2. Department of Rheumatology, Oslo University Hospital, Oslo, Norway

Department of Cariology and Gerodontology. Faculty of Dentistry, University of Oslo, Oslo, Norway.

1. Department of Ophthalmology, Oslo University Hospital, Norway.
2. Department of Oral Biology, Faculty of Dentistry, University of Oslo, Oslo, Norway
3. Department of Medical Biochemistry, Oslo University Hospital, Norway

#

**Supplementary material**

***Appendix 1* Medication use in non-SS and pSS patients and in controls**

|  | **Non-SS (n=22)** | **%** | **pSS (n=60)** | **%** | **Control**  **(n=43)** | **%** | p value  ANOVA |
| --- | --- | --- | --- | --- | --- | --- | --- |
| Allergy medicines | 5 | 23 | 12 | 20 | 5 | 12 | 0.335 |
| Antibiotics | 0 | 0 | 0 | 0 | 0 | 0 |  |
| Antidepressants | 2 | 9 | 5 | 8 | 2 | 4.6 | 0.123 |
| Anticholinergics | 0 | 0 | 0 | 0 | 0 | 0 |  |
| Antipsychotics | 0 | 0 | 0 | 0 | 0 | 0 |  |
| Asthma medicines | 1 | 4.50 | 2 | 3 | 1 | 2 | 0.877 |
| Drugs affecting bone structure | 0 | 0 | 2 | 3 | 0 | 0 | 0.304 |
| Hypnotics and sedatives | 6^a,b^ | 27 | 3 | 5 | 1 | 2 | 0.001 |
| Anticoagulants | 2 | 9 | 5 | 8 | 0 | 0 | 0.123 |
| Diabetes medicine | 1 | 4.5 | 1 | 1.6 | 0 | 0 | 0.396 |
| Cardiovascular medicines inc. statins | 5 | 23 | 14^c^ | 23 | 1 | 2 | 0.003 |
| Hormones inc. thyroid medicines | 3 | 14 | 11 | 18 | 3 | 7 | 0.179 |
| Chemotherapeutics | 0 | 0 | 1 | 1.6 | 0 | 0 | 0.556 |
| Steroids | 0 | 0 | 1 | 1.6 | 0 | 0 | 0.556 |
| Food supplements/natural medicines | 7 | 32 | 19 | 32 | 14 | 33 | 0.961 |
| Analgetics | 11^b^ | 50 | 19^c^ | 32 | 1 | 2 | <0.001 |
| Antacids | 0 | 0 | 8^c^ | 13 | 0 | 0 | 0.005 |
| Plaquenil | 1 | 4.5 | 12^c^ | 20 | 0 | 0 | 0.001 |
| Salagen | 0 | 0 | 1 | 1.6 | 0 | 0 | 0.550 |
| Eyedrops | 4 | 18 | 11 | 18 | 0 | 0 | 0.006 |
| Others | 7 | 32 | 20^c^ | 33 | 3 | 7 | 0.002 |

^a^ Significant difference between non-SS and pSS, p<0.05

^b^ Significant difference between non-SS and controls, p<0.05

^c^ Significant difference between pSS and controls, p<0.05

***Appendix 2* Results of correlation analyses between GHRQoL and oral/ocular dryness in pSS, non-SS sicca and healthy control groups**

| Parameters | r | *p* value |
| --- | --- | --- |
| pSS | | |
| Physical functioning and SXI | -0.331 | =0.013 |
| Physical functioning and OSDI | -0.353 | =0.008 |
| Role physical and SXI | -0.416 | =0.001 |
| Role physical and OSDI | -0.460 | <0.001 |
| General health and SXI | -0.408 | =0.002 |
| Bodily pain and OSDI | -0.338 | =0.003 |
| Vitality and SXI | -0.322 | =.015 |
| Vitality and OSDI | -0.346 | =0.009 |
| Social functioning and SXI | -0.414 | =0.002 |
| Social functioning and OSDI | -0.332 | =0.012 |
| Role emotional and SXI | -0.267 | =0.046 |
| Role emotional and OSDI | -0.377 | =0.004 |
| PCS and SXI | -0.406 | <0.001 |
| PCS and OSDI | -0.450 | <0.001 |
| MCS and SXI | -0.325 | <0.001 |
| MCS and OSDI | -0.287 | <0.001 |
| Non-SS |  |  |
| Role physical and vital staining | -0.576 | =0.006 |
| Controls |  |  |
| Physical functioning and OSDI | -0.338 | =0.038 |
| Physical functioning and tear osmolarity | -0.344 | =0.04 |
| Vitality and SXI | -0.798 | =0.032 |
| Social functioning and OSDI | -0.471 | =0.003 |
| Social functioning and candida score | 0.386 | =0.017 |
|  |  |  |

***Appendix 3* Results of correlation analyses between OHRQoL and oral/ocular dryness in pSS, non-SS sicca and healthy control groups**

| Parameters | r | *p* value |
| --- | --- | --- |
| pSS | | |
| OHIP Q1and SXI | 0.345 | =0.008 |
| OHIP Q1 and candida score | 0.360 | =0.01 |
| OHIP Q1 and CODS | 0.274 | =0.036 |
| OHIP Q1 and UWS | -0.294 | =0.024 |
| OHIP Q1 and SWS | -0.269 | =0.039 |
| OHIP Q1 and tear osmolarity | 0.404 | =0.005 |
| OHIP Q3 and SXI | 0.383 | =0.003 |
| OHIP Q3 and OSDI | 0.360 | =0.006 |
| OHIP Q4 and SXI | 0.462 | <0.001 |
| OHIP Q4 and tear osmolarity | 0.437 | =0.002 |
| OHIP Q5 and SXI | 0.331 | =0.012 |
| OHIP Q6 and SXI | 0.484 | <0.001 |
| OHIP Q7 and UWS | -0.265 | =0.044 |
| OHIP Q8 and SXI | 0.338 | =0.011 |
| OHIP Q10 and CODS | 0.264 | =0.044 |
| OHIP Q10 and OSDI | 0.277 | =0.037 |
| OHIP Q10 and tear osmolarity | 0.427 | =0.001 |
| OHIP 11 and SXI | 0.388 | =0.003 |
| OHIP 11 and UWS | -0.267 | =0.043 |
| OHIP 11 and OSDI | 0.276 | =0.039 |
| OHIP 12 and SXI | 0.397 | =0.002 |
| OHIP 12 and candida score | 0.306 | =0.029 |
| OHIP 12 and UWS | -0.315 | =0.015 |
| OHIP 12 and OSDI | 0.270 | =0.043 |
| OHIP 12 and tear osmolarity | 0.311 | =0.035 |
| OHIP SUM and SXI | 0.549 | <0.001 |
| OHIP SUM and candida score | 0.792 | =0.05 |
| OHIP SUM and UWS | -0.267 | =0.43 |
| OHIP SUM and tear osmolarity | 0.298 | =0.045 |
| Non-SS |  |  |
| OHIP Q1 and UWS | -0.662 | =0.001 |
| OHIP Q1 and SWS | -0.641 | =0.001 |
| OHIP Q2 and vital staining | -0.447 | =0.048 |
| OHIP Q3 and SWS | -0.436 | =0.042 |
| OHIP Q3 and OSDI | 0.563 | =0.006 |
| OHIP Q4 and UWS | -0.440 | =0.041 |
| OHIP Q4 and SWS | -0.624 | =0.002 |
| OHIP Q4 and vital staining | -0.462 | =0.035 |
| OHIP Q8 and SWS | -0.482 | =0.023 |
| OHIP Q8 and OSDI | 0.427 | =0.047 |
| OHIP Q8 and vital staining | -0.604 | =0.004 |
| OHIP Q9 and vital staining | -0.539 | =0.012 |
| OHIP 10 and vital staining | -0.536 | =0.012 |
| OHIP 12 and SWS | -0.608 | =0.003 |
| OHIP 12 and vital staining | -0.515 | =0.017 |
| OHIP 13 and vital staining | -0.463 | =0.04 |
| OHIP SUM and tear OSDI | 0.596 | =0.003 |
| OHIP SUM and TFBUT | 0.491 | =0.02 |
| OHIP SUM and vital staining | -0.725 | <0.001 |
| Controls |  |  |
| OHIP Q1 and tear osmolarity | 0.326 | =0.042 |
| OHIP Q2 and Schirmer test | -0.415 | =0.01 |
| OHIP Q6 and Schirmer test | -0.433 | =0.007 |
| OHIP Q8 and Schirmer test | -0.466 | =0.003 |
| OHIP 9 and Schirmer test | -0.342 | =0.036 |
| OHIP 11 and UWS | 0.304 | =0.05 |
| OHIP 11 and Schirmer test | -0.334 | =0.041 |
| OHIP 12 and UWS | 0.304 | =0.05 |
| OHIP 12 and Schirmer test | -0.334 | =0.041 |
| OHIP 13 and Schirmer test | 0.330 | =0.043 |
